# Novel Approaches for Early Detection of Chronic Insomnia Among Older Primary Care Patients

**DOI:** 10.1093/geroni/igaf122.3101

**Published:** 2025-12-31

**Authors:** Jaime Hughes, Corey Obermiller, Laura Jakiela, Case Peters, Lindsay Munn, Deepak Palakshappa

**Affiliations:** Wake Forest University School of Medicine, Winston-Salem, North Carolina, United States; Atrium Health Wake Forest Baptist Medical Center, Winston-Salem, North Carolina, United States; Wake Forest University School of Medicine, Winston-Salem, North Carolina, United States; Wake Forest University School of Medicine, Winston-Salem, North Carolina, United States; Wake Forest University School of Medicine, Winston-Salem, North Carolina, United States; Wake Forest University School of Medicine, Winston-Salem, North Carolina, United States

## Abstract

Sleep disturbance is common among older adults, contributing to social, cognitive, and functional impairments when untreated. Given sleep disturbance is not routinely addressed within primary care, novel methods for its early identification are needed. In partnership with five primary care clinics belonging to a multistate healthcare system, older adults at risk for insomnia, defined by presence of prespecified comorbidities, were identified from the electronic health record and sent a validated sleep screener. Screener domains included sleep schedule, nighttime sleep disturbances, daytime consequences of poor sleep, overall sleep quality, and history of talking with a provider about sleep. Respondents meeting clinical criteria for insomnia were contacted for a telephone interview and invited to participate in additional research activities (interview, co-design, or CBTI treatment). Of 3455 surveys distributed, 446 older adults (mean age: 75(7.2) years, 37% Male, 67% White, 42% pre-frail, 23% poor health) returned a completed survey. One-quarter of respondents screened positive for chronic insomnia (N = 117); yet, only 44 (38%) had an EHR-documented insomnia diagnosis. When asked to describe their sleep in subsequent interviews (N = 94), older adults endorsed a range of symptoms including nighttime sleep complaints (N = 39 (40%), difficulty falling or staying asleep) and negative impacts on daytime function (N = 54 (56%)). These findings indicate insomnia is prevalent yet underdiagnosed and variable in its symptom profile. Patients’ preferences for integrating insomnia screening into primary care included completing a screener via the patient portal prior to a scheduled encounter, tips on communicating sleep problems (e.g., nighttime vs. daytime symptoms), and age-friendly sleep recommendations.

